# Inflammation in Coronary Artery Ectasia Compared to Atherosclerosis

**DOI:** 10.3390/ijms19102971

**Published:** 2018-09-29

**Authors:** Ertan Yetkin, Selcuk Ozturk, Gulay Imadoglu Yetkin

**Affiliations:** 1 Private Yenisehir Hospital Division of Cardiology, Mersin 33100, Turkey; 2 Ankara Research and Training Hospital, Department of Cardiology, Ankara 06310, Turkey; selcukozturk85@hotmail.com; 3 International Medical Center IMC Hospital, Division of Microbiology, Mersin 33100, Turkey; gulayyetkin03@hotmail.com

Dear Editor,

We have read with great enthusiasm the article recently published by Boles et al. [1]. Briefly, they compared the systemic immunoinflammatory status in patients with coronary artery ectasia (CAE) with the cytokine milieu in healthy control subjects and patients with coronary artery disease (CAD). CAE patients showed increased systemic levels of INF-γ, TNF-α, IL-1β, IL-6, and IL-8 and lower IL-2 and IL-4 than control subjects and patients with CAD. They have also commented that these increased inflammatory responses are likely the result of macrophage-mediated cytokine production.

Although CAE has increasingly gained interest in the literature over the last decades, the pathophysiologic mechanism of CAE still remains to be elucidated. Frequent coexistence of CAE with CAD is the likely reason for the lack of knowledge regarding the exact mechanism of CAE. However, distinct differences between CAE and CAD have been documented in the literature in terms of clinical course and pathophysiologic steps and biomarkers [2,3,4,5,6]. The most promising difference is the increased inflammatory status in patients with CAE compared to those with normal coronary arteries and CAD, as demonstrated by Boles et al. Increased cellular activation molecules such as intercellular adhesion molecule (ICAM), vascular cell adhesion molecule (VCAM), E-selectin, C-reactive protein, and activation markers of macrophages have already been documented as indicating increased inflammation in CAE compared to CAD [7,8,9,10]. What needs to be figured out is the underlying mechanism of increased inflammatory state in CAE. Regarding the main pathophysiologic mechanism of CAE, vascular wall abnormality or destruction is the key process of the disease. Albeit difficult to prove yet, vascular wall abnormality may originate from hereditary factors or infectious etiology resulting in improper extracellular matrix remodeling through an inflammatory process. So far, Boles et al. [1] has added another proof against atherosclerosis in the pathophysiology of CAE. Moreover, given the common coexistence of CAE with other arterial and venous aneurismal dilatations [2,3,11], it is time to consider CAE as a manifestation of systemic vascular wall abnormalities rather than a localized disease of the heart. Likewise, Juvonen et al. [12] has already documented increased levels of IL-1β, IL-8 TNF-α, and interferon-gamma in patients with abdominal aortic aneurysm, which is another example of a dilating vascular disease. In conclusion, coronary artery ectasia or its coexistence, namely, dilating vascular disease, should be investigated individually to figure out the main underlying mechanisms or etiologies rather than focusing on atherosclerosis itself.
